# 6-Hydroxyflavanone treats anxiety and chemotherapy-induced neuropathy in Sprague–Dawley rats

**DOI:** 10.3389/fphar.2024.1486918

**Published:** 2024-12-18

**Authors:** Shehla Akbar, Fazal Subhan, Rida Qamar, Aroosha Akbar, Naila Shahbaz, Muhammad Aamir, Nayyer Siddique, Salman Ul Islam

**Affiliations:** ^1^ Department of Pharmacy, CECOS University of IT and Emerging Sciences, Peshawar, Pakistan; ^2^ Armed Forces Institute of Dentistry, Army Medical College, Rawalpindi, Pakistan; ^3^ Department of Physical Therapy, Northwest College of Rehabilitation Sciences, Northwest Institute of Health Sciences, Peshawar, Pakistan; ^4^ Department of Pharmacy, Sarhad Institute of Science and Information Technology, Peshawar, Pakistan; ^5^ Department of Pharmacy, The University of Lahore, Lahore, Pakistan; ^6^ Department of Molecular Biology and Genetics, Khyber Medical University, Peshawar, Pakistan; ^7^ Department of Pharmacy, International Institute of Science, Arts, and Technology (IISAT), Gujranwala, Pakistan

**Keywords:** chemotherapy, 6-hydroxyflavanone, neurotoxicity, anxiety, cancer

## Abstract

**Background:**

Cancer remains a predominant cause of death worldwide. The advent of effective chemotherapy has enormously decreased the mortality rate and increased the life expectancy of cancer patients. However, the adverse effects allied with chemotherapy contribute to the development of neurotoxicity, anxiety, and depression.

**Objective:**

The dual effects of a novel flavanone, 6-hydroxyflavanone (6-HF), were investigated in treating chemotherapy-induced neuropathy along with anxiolytic propensity.

**Methods:**

The anti-neuropathy propensity of 6-HF was evaluated utilizing the cisplatin-induced neuropathy (CIN) model, whereas its anxiolytic activity was evaluated utilizing anxiety models, such as the elevated plus maze test (EPM), the staircase test, and the open-field paradigm.

**Result:**

Cisplatin administration induced static and dynamic allodynia in the rats. Concomitant administration of 6-HF and cisplatin for four successive weeks remarkably reduced the chemotherapy-induced mechanical allodynia, evident from an elevation in the paw withdrawal threshold (PWT) and paw withdrawal latency (PWL). The anxiolytic-like activity of 6-HF in the EPM apparatus was confirmed by the increased number of entries in the open arm and time spent at the central platform, which was further confirmed by the enhanced head-dipping frequency in the same assay. A decrease in rearing behavior of the mice without suppression of the steps ascended further assured the anxiolytic-like potential of 6-HF. Additionally, the animals under investigation spent more time at the intersection of the open-field apparatus, further confirming the anxiolytic potential of 6-HF.

**Conclusion:**

6-HF might be considered a potential therapeutic agent for counteracting two common adverse effects of chemotherapy, neurotoxicity and anxiety.

## 1 Introduction

Cancer, or abnormal cell proliferation, is the main cause of death worldwide, with a mortality rate of 8.7 million (Bray et al., 2018). Although much research is investigating appropriate treatments, including chemotherapy, the usage of chemotherapy is restricted due to the adverse effects levied by anticancer agents. Neurotoxicity resulting in peripheral neuropathic pain remains one of the most debilitating adverse effects associated with the use of chemotherapeutic agents like platinum analogs. Despite the significant therapeutic potency of platinum analogs in treating various types of cancers, neurotoxicity limits their use as it leads to a compromise in patient’s quality of life and poor compliance (Djordjević et al., 2023). This neurotoxicity is related to the accumulation of cisplatin analogs in the dorsal root ganglia, inducing neuronal dysfunction and apoptosis. This damage to the somatosensory system manifests as hyperalgesia, allodynia, paresthesia, or numbness in the lower extremities of the body (Ibrahim and Ehrlich, 2019).

The neuropathy is often accompanied by anxiety, further compromising the patient’s compliance. Prolonged anxiety leads to severe depression, forcing the patient to take anxiolytic/anti-depressant drugs. The adverse effects of the anti-depressants are well-documented, including but not limited to physical and chemical effects, cardiovascular adverse reactions, gastrointestinal problems, sleep disturbance, apathy, and fatigue (Tang and Li, 2020). These side effects can further impact cancer treatment adherence, quality of life, and patient response to pharmacotherapy. Consequently, a patient already suffering from the deleterious consequences of cancer finds it more difficult to indulge in polypharmacy, which not only compromises the cancer management plan but also leads to premature treatment discontinuation (Nightingale et al., 2017). The development of anxiety-like behavior in rat models has been reported, suggesting that chronic neuropathic pain results in the emergence of anxiety, which further exaggerates the pain perception (Röska, 2009). However, both parameters have not yet been concomitantly reported in a single study. There is an obvious need for drugs targeting more than one domain and thus treating multiple complications allied with chemotherapy. In this regard, flavonoids emerged as a class of drugs known for several pharmacological activities, including anticancer, anxiolytic, anti-nociceptive, anti-inflammatory, and antioxidant properties (Zhou et al., 2019; Shukla et al., 2019). 6-Hydroxyflavanone (6-HF) was previously investigated for anti-neuropathy activity in a diabetes-induced neuropathy model. It was confirmed via *in silico*, *in vitro*, and *in vivo* testing that 6-HF inhibited the COX-2 and 5-LOX enzymes. Moreover, the GABAergic and opioidergic actions of the compound were elucidated via computational studies and confirmed by the administration of antagonists like PTZ and naloxone (Akbar et al., 2023). In the current study, 6-HF was co-administered with a platinum analog (cisplatin) to analyze its protective potential against neuropathy in the cisplatin-induced neuropathy (CIN) model, together with its anxiolytic activity in rat models of anxiety. The dual effect of 6-HF in treating both the neuropathic pain and anxiety in patients being treated with chemotherapy could provide a therapeutic alternative to polypharmacy, limiting the advent of multiple adverse effects of anti-neuropathic pain and anti-depressant drugs that ultimately result in patient non-compliance.

## 2 Materials and methods

### 2.1 Chemical and drug supplies

Analytical-grade chemicals and drugs were used in this research. 6-HF (>95%), cisplatin (≥99.9%), and gabapentin (99%) were purchased from Merck, United States. Diazepam (10 mg/2 mL) was obtained from Roche, Pakistan. The vehicle for 6-HF consisted of 1% Tween 80, 5% DMSO, and 94% normal saline.

### 2.2 Animals

Male Sprague–Dawley rats (150–250 g; age 8 weeks), bred in the animal house and bioassay laboratory of the Department of Pharmacy, University of Peshawar, were used. The animals were housed under conditions of constant temperature (22°C) and a light/dark cycle of 12 h. They were given recommended animal food and clean water as needed. A total of 126 rats were used in this study. The study was conducted according to the animal maintenance and use guidelines of the Scientific Procedures Act, 1986, following the approval from the University of Peshawar Ethical Board under the Registration number: 08/EC-15/Pharm on 20 February 2015.

### 2.3 Cisplatin-induced neuropathy

To induce neuropathy, the study was conducted in male Sprague–Dawley rats (150–250 g; age of 8 weeks) for 4 weeks (28 days). The rats were administered cisplatin with an intraperitoneal dose of 3.00 mg/kg once per week as per the previously defined procedure (Han et al., 2014). To prevent renal damage, 2 mL of normal saline was administered subcutaneously (s.c.) to the animals prior to the administration of cisplatin (Authier et al., 2003). The standard drug, gabapentin, at a dose of 75.0 mg/kg, and 6-HF, at doses of 15.0 mg/kg, 30.0 mg/kg, and 60.0 mg/kg, were administered intra-peritoneally to the animals 30 min prior to the cisplatin injection. These doses of 6-HF were selected on the basis of previous studies conducted on the same compound in different pain models (Akbar et al., 2023). Gabapentin (75.0 mg), as the first line of treatment in neuropathic pain studies beside TCAs and SNRIs, was considered the standard drug (Thouaye and Yalcin, 2023), as it has already been used by researchers studying novel compounds for treating neuropathic pain in rodent models of neuropathy (Ahmad et al., 2021; Shahid et al., 2020). The distribution of animals into different groups is given in Table 1.

**TABLE 1:** The table shows the dose administration scheme for the induction of neuropathy by cisplatin (3.00 mg/kg) and the scheme for the co-administration of 6-hydroxyflavanone at doses of 15.0 mg/kg, 30.0 mg/kg, and 60.0 mg/kg in the male Sprague Dawley rat model of CIN (n = 6).

Group (male Sprague–Dawley rats, n = 6)	Treatment	Dose
Group-i	Vehicle	1.00 mL/kg/week, intraperitoneal
Group-ii	Cisplatin	3.00 mg/kg/week, intraperitoneal
Group-iii	Gabapentin plus cisplatin	75.0 mg/kg/week, intraperitoneal, plus 3.00 mg/kg/week, intraperitoneal
Group-iv	6-HF plus cisplatin	15.0 mg/kg/week, intraperitoneal, plus 3.00 mg/kg/week, intraperitoneal
Group-v	6-HF plus cisplatin	30.0 mg/kg/week, intraperitoneal, plus 3.00 mg/kg/week, intraperitoneal
Group-vi	6-HF plus cisplatin	60.0 mg/kg/week, intraperitoneal, plus 3.00 mg/kg/week, intraperitoneal

#### 2.3.1 Behavioral analysis of allodynia in CIN

The standard protocol, involving a von Frey hair apparatus, was employed for the evaluation of both static and dynamic allodynia induced by the injections of cisplatin (Stoelting, Wood Dale, Illinois, United States). In brief, for the assessment of static allodynia, the up–down method depicting the application of the von Frey filaments (0.4 g, 0.70 g, 1.20 g, 2.00 g, 3.63 g, 5.50 g, 8.50 g, and 15.10 g; starting with a 2.0 g force) perpendicularly to the plantar region of the right hind paw (cutoff: 6 s) was adopted, and the response of the rats was observed accordingly (Chaplan et al., 1994). The lifting of the paw, termed the paw withdrawal threshold (PWT; g), was reflected as the endpoint response, which was followed by the application of the higher force of the filaments. In this way, four readings were taken after the first positive response (increasing the force from 2 g) or five successive negative responses (decreasing the force below 2 g). The behavioral assessment was performed on the same animals randomly distributed into different groups, as mentioned in Table 1.

The dynamic allodynia was evaluated via a cotton bud. The same region of the rat’s hind paw was gently stroked with the cotton bud, and the lifting or licking of the paw was taken as a positive response. The time taken to elicit the positive response was considered paw withdrawal latency (PWL; s), and a time of 15 s was selected as the cutoff time.

### 2.4 Behavioral analysis in anxiety models

The behavioral analysis was conducted during the light phase (8:00 a.m. to 2:00 p.m.) in a weakly irradiated room with a red light source. The rats under study were housed in separate cages (six rats per cage). The animals were brought to the experimental laboratory 1 h prior to the application of the standard procedure. 6-HF was administered in doses of 15.0 mg/kg, 30.0 mg/kg, and 60.0 mg/kg (i.p.), whereas diazepam was administered at a dose of 2.00 mg/kg (i.p.), 30 min before the behavioral analysis. These tests were performed on different male rats divided into subgroups because the rationale of this study was to evaluate the anxiolytic propensity of 6-HF compared to the commonly used anxiolytic agent (diazepam). The 2.0 mg/kg dose of the standard diazepam was selected because the anxiolytic effect of diazepam at this dose is related to the increase in GABA levels causing anxiolysis, whereas a higher dose of 5.0 mg/kg causes sedation (Walia et al., 2019). The doses of 6-HF were selected on the basis of previously reported studies on similar synthetic flavonoids (Akbar et al., 2017). The assessment of neuropathy and anxiolytic actions of 6-HF in the same animal models could be a future prospect for this study.

#### 2.4.1 Elevated plus maze test

An elevated plus maze apparatus was utilized to assess the anxiolytic behavior in male rats after the administration of the standard and the test drug. In brief, the apparatus was equipped with two open arms and two closed arms having a common intersection (Macri et al., 2002). After the completion of the habituation time, the rats were randomly divided into five groups with a sample size of n = 6. The animals of group-i were considered the control group and were injected with the vehicle. Group-ii was considered the standard control group and was administered with diazepam (2.0 mg/kg), whereas groups-iii, iv, and v were administered with the selected doses of 6-HF (15.0 mg/kg, 30.0 mg/kg, and 60.0 mg/kg). Thirty minutes later, the rats were positioned on the intersection of the elevated plus maze apparatus, heading toward the open arms. The behavioral factors, including spatiotemporal parameters such as the open/closed arm entries and time spent in the open arm and at the intersection, were recorded for 5 min. The ethological factors included the recording of head-dipping frequency and rearing for a 5-min period (Pellow et al., 1985; Rodgers et al., 1995). The Cat’s Eye IR camera was used to record the behavioral sessions. The elevated plus maze apparatus was cleaned thoroughly using wet tissues.

#### 2.4.2 Staircase test

The staircase model comprised five steps. The rats were habituated to the behavioral analysis laboratory 1 h prior to the experiment and were randomly divided into five groups with a sample size of n = 6. Group-i was considered the control group and was injected with a vehicle. Group-ii was considered the standard control group and was administered with diazepam (2.0 mg/kg; i. p.), whereas groups-iii, iv, and v were administered with 6-HF (15.0 mg/kg, 30.0 mg/kg, and 60.0 mg/kg; i. p.). The rats were placed on the staircase apparatus 30 min post administration of the drugs. They were observed for the number of steps ascended and the frequency of rearing (Simiand et al., 1984). The rats placing all four paws on a step qualified as ascending one step.

#### 2.4.3 Open-field test

The open-field kit consisted of a case divided into four equal quadrants and monitored using a Cat’s Eye IR camera. The rats were randomly divided into five groups in the same manner as in the other two tests. They were positioned at the central point of the open-field apparatus 30 min post-administration of the control, diazepam (2.0 mg/kg), and 6-HF (15.0 mg/kg, 30.0 mg/kg, and 60.0 mg/kg). The rats were observed for the number of lines crossed and time spent at the intersection of the box for a 5-min duration (Hall, 1934).

## 3 Data analysis

The statistical analysis of the data obtained was carried out with GraphPad Prism 5.0. The results were expressed as the mean ± SEM. One-way ANOVA with *post hoc* Dunnett’s test or two-way ANOVA with *post hoc* Bonferroni’s test was applied over the data as needed. A *p* < 0.05 was considered to be significant.

## 4 Results

### 4.1 Effect of 6-HF and gabapentin in allodynia

**FIGURE 1:**
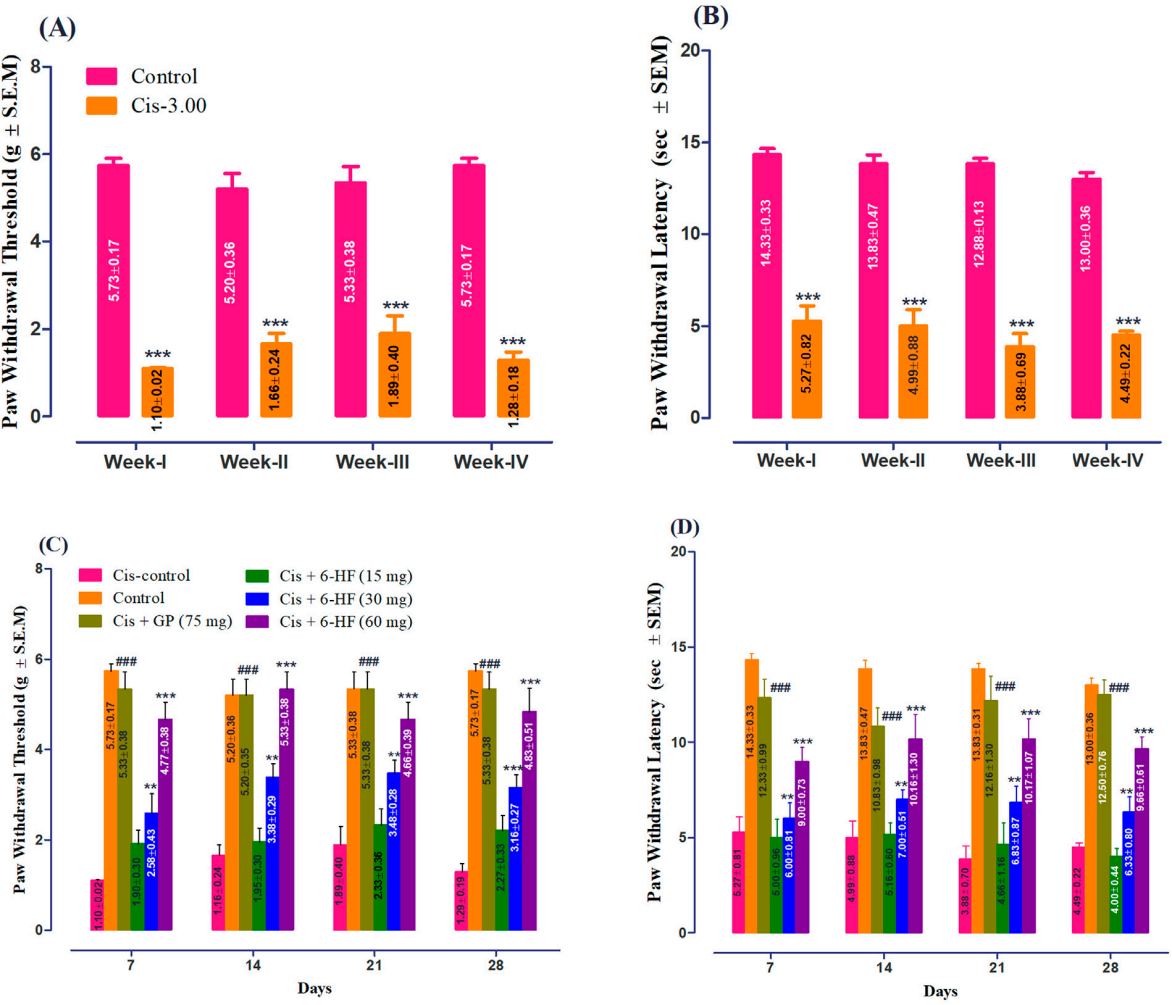
Induction of peripheral neuropathy by weekly administration of cisplatin at a dose of 3.00 mg/kg for 4 weeks with respect to **(A)** paw withdrawal threshold (PWT; mean ± SEM) and **(B)** paw withdrawal latency (PWL; mean ± SEM) in male Sprague–Dawley rats. ***p < 0.001 compared to vehicle-treated control animals. Two-way repeated measure ANOVA *post hoc* Bonferroni’s analysis (n = 6 rats per group). Effect of co-administration of 6-HF at doses of 15 mg/kg, 30 mg/kg, and 60 mg/kg and the positive control gabapentin (GP) at a dose of 75 mg/kg on the expression of mechanical-static/dynamic allodynia induced by the administration of cisplatin (3.0 mg/kg; Cis-3) for four consecutive weeks in male Sprague–Dawley rats. **(C)** Effect of 6-HF and GP on the paw withdrawal threshold (PWT in g; static allodynia). **(D)** Effect of 6-HF and GP on the paw withdrawal latency (PWL in sec; dynamic allodynia). ^###^ *p* < 0.001 compared to vehicle (Veh)-treated animals. **p < 0.01 and ***p < 0.001 compared to cisplatin-treated control animals. Two-way repeated measure ANOVA *post hoc* Bonferroni’s analysis (n = 6 rats per group).

The study was conducted in male Sprague–Dawley rats to evaluate the neuroprotective effects of 6-HF against cisplatin-induced peripheral neuropathy. Both drugs were administered concomitantly. The rats administered with a single injection of cisplatin at a dose of 3.00 mg/kg for a period of 4 weeks developed significant static allodynia [decreased PWT; 1.485 ± 0.18 g; t (6) = 17.78, *p* < 0.0001] and dynamic allodynia [decreased PWL; 4.665 ± 0.305 g; t (6) = 22.06, *p* < 0.0001] when compared to the control group, as shown in Figures 1A, B.

6-HF exhibited a significant anti-nociception against static allodynia at doses of 30.0 mg/kg and 60.0 mg/kg [time (F (3,120) = 0.83; *p* = 0.481), treatment (F (5,120) = 105.87; *p* < 0.0001), and interaction (F (15,120) = 0.73; *p* = 0.745)] when compared to the cisplatin-control group (Figure 1C). Moreover, the standard gabapentin demonstrated a protective effect against cisplatin-induced neuropathy, signified by the increase in PWT (F (5,18) = 141.2; *p* < 0.0001).

Similarly, in the case of dynamic allodynia, the co-administration of 6-HF significantly protected against nociception at doses of 30.0 mg/kg and 60.0 mg/kg [time (F (3,120) = 0.23; *p* = 0.87), treatment (F (5,120) = 90.09; *p* < 0.0001), and interaction (F (15,120) = 0.58; *p* = 0.88)] compared to the cisplatin-treated group (Figure 1D). Moreover, the standard gabapentin demonstrated a protective effect against the cisplatin-induced neuropathy signified by the increase in PWL (F (5,18) = 173.9; *p* < 0.0001).

### 4.2 Effect of 6-HF and diazepam on anxiety in the EPM

**FIGURE 2:**
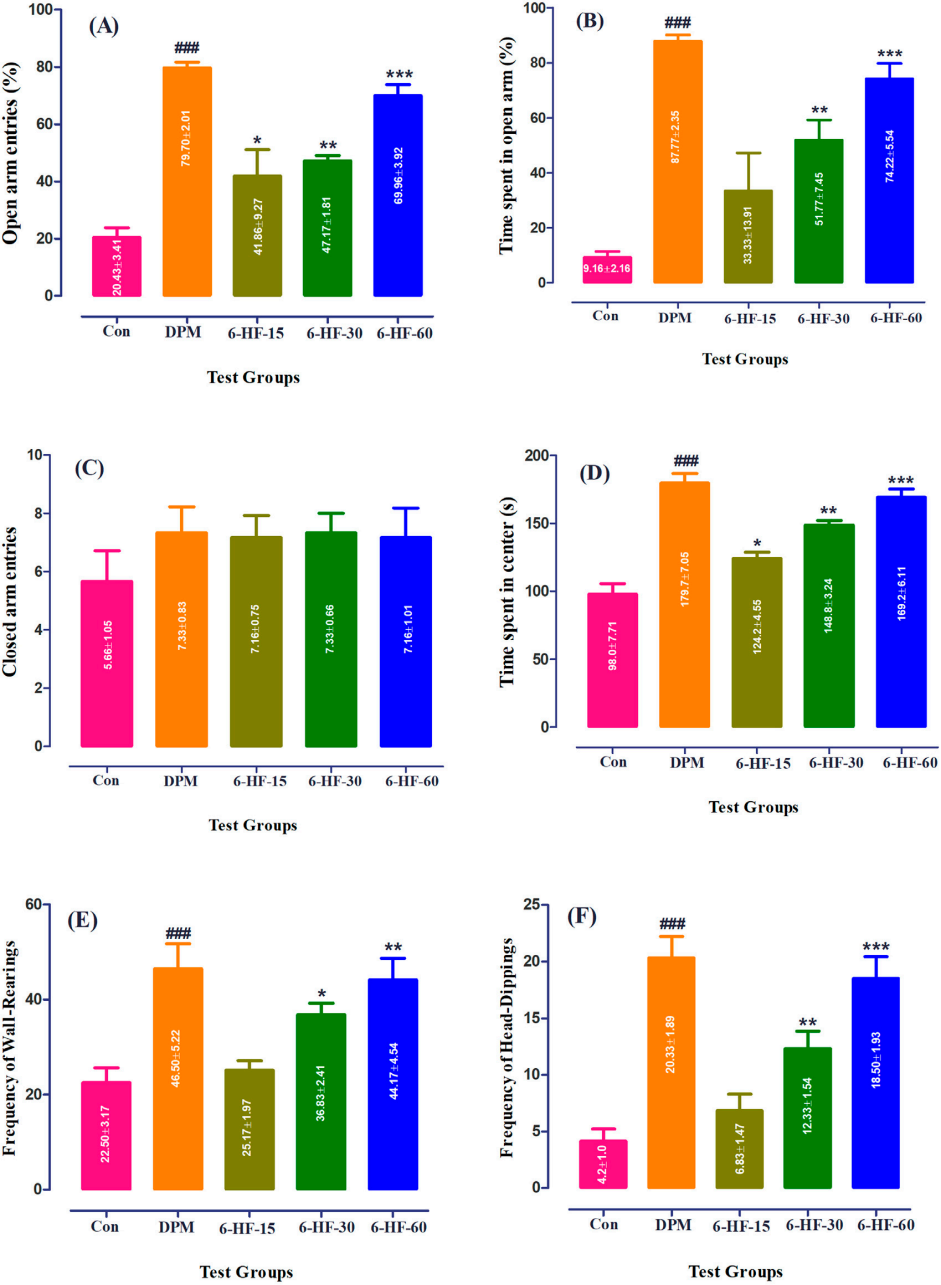
Effect of 6-HF at doses of 15 mg/kg (6-HF-15), 30 mg/kg (6-HF-30), and 60 mg/kg (6-HF-60) and diazepam (DPM) at a dose of 2 mg/kg in male Sprague–Dawley rats on the **(A)** open-arm entries, **(B)** time spent in the open arms, **(C)** frequency of wall-rearing, **(D)** frequency of head-dipping, **(E)** closed-arm entries, and **(F)** the time spent at the center of apparatus in the elevated plus maze model of anxiety. One-way ANOVA *post hoc* Dunnett’s analysis (n = 6 rats per group; mean ± SEM).

Both the spatiotemporal and ethological parameters were assessed in male rats to determine the anxiolytic potential of 6-HF in the EPM model. 6-HF (15.0 mg/kg, 30.0 mg/kg, and 60.0 mg/kg) and the standard diazepam (2.00 mg/kg) significantly elevated the open-arm entries [F (4,25) = 23.0; *p* ˂ 0.0001], time duration dedicated to the open arms [F (4,25) = 17.05; *p* ˂ 0.0001], and the time duration dedicated to the center [F (4,25) = 31.21; *p* ˂ 0.0001] of the apparatus (Figures 2A, B, D) compared to the vehicle-administered group. Moreover, the animals showed insignificant changes in the closed-arm entries [F (4,25) = 0.64; *p* = 0.6335], as shown in Figure 2C.

The ethological factors showed an increase in rearing behavior [F (4,25) = 8.732;, *p* ˂ 0.0001] and head-dipping [F (4,25) = 19.41; *p* ˂ 0.0001] by the rats treated with 6-HF (the tested doses of 30.0 mg/kg and 60.0 mg/kg) and diazepam (Figures 2E, F).

### 4.3 Effect of 6-HF as an anxiolytic agent in the staircase test

**FIGURE 3:**
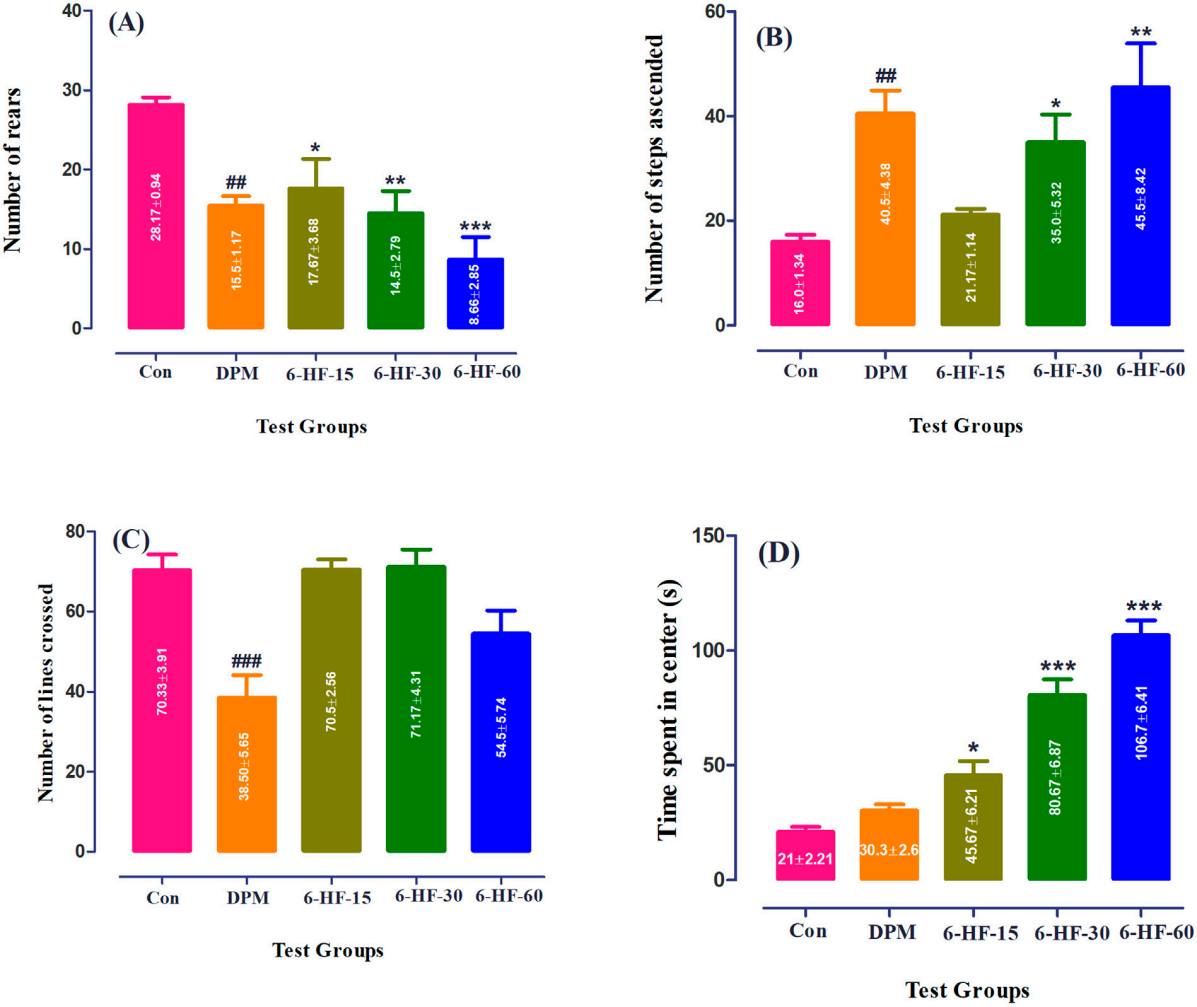
Effect of 6-HF at doses of 15 mg/kg (6-HF-15), 30 mg/kg (6-HF-30), and 60 mg/kg (6-HF-60) and diazepam (DPM) at a dose of 2 mg/kg in Sprague–Dawley rats on the **(A)** number of steps ascended, **(B)** number of wall-rearing instances in the staircase model, **(C)** number of lines crossed, and **(D)** time spent in center of the apparatus in the open-field model of anxiety. One-way ANOVA *post hoc* Dunnett’s analysis (n = 6 rats per group; mean ± SEM).

The Sprague–Dawley rats treated with 6-HF at doses of 15.0 mg/kg, 30.0 mg/kg, and 60.0 mg/kg and diazepam showed a significant decrease [F (4,25) = 7.993; *p* = 0.0003] in the number of rears in the staircase paradigm compared to the vehicle-administered group (Figure 3A), whereas the number of steps ascended were increased significantly [F (4,25) = 6.544; *p* = 0.001] at the doses of 30.0 mg/kg (*p* < 0.05) and 60.0 mg/kg (*p* < 0.01) (Figure 3B).

### 4.4 Effect of 6-HF as an anxiolytic agent in the open-field test

In this test, 6-HF at the tested doses did not show an effect on the locomotion of the Sprague–Dawley rats when compared to the control group, whereas diazepam showed a significant decrease [F (4.25) = 9.838; *p* < 0.0001] in the locomotion at a dose of 2.00 mg/kg (Figure 3C). However, the time duration dedicated to the intersection of the open-field apparatus by the animals was significantly increased [F (4.25) = 46.43; *p* < 0.0001] at all doses (15.0 mg/kg, 30.0 mg/kg, and 60.0 mg/kg) compared to the vehicle-administered group (Figure 3D). Moreover, the standard drug diazepam showed no effect on the time spent at the intersection of the apparatus.

## 5 Discussion

Cancer was considered an incurable disease, causing massive mortality in the late 1990s (Blackadar, 2016). The advancement in chemotherapeutic agents not only improved the life expectancy of patients fighting cancer but also provided hope to the scientific community. Although they are quite effective in treating the disease, chemotherapeutic agents bring several adverse effects along with their cure (Choi et al., 2023). Much research is underway, but there is still a dire need for the development of therapeutic agents that provide protection against the deleterious adverse effects of these agents without compromising their affectivity.

In this investigation, 6-HF was observed to protect the rats against the peripheral neuropathy induced by cisplatin. 6-HF also showed an anxiolytic-like activity when evaluated in anxiety models. Neuropathy and anxiety are considered the most common and harmful adverse effects associated with the use of cisplatin as a chemotherapeutic agent (McNeish et al., 2021; Hung et al., 2021). Cisplatin-induced neuropathy is attributed to the accumulation of platinum adducts within the neuronal cells, which interrupts the proper functioning of the cells. The dorsal root ganglia are affected mostly due to the lack of a blood–brain barrier in this region, causing sensory neuropathy manifested as paresthesias, allodynia, and numbness. It has been reported that approximately 70% of the patients prescribed platinum analogs develop peripheral neuropathy that starts 1-week post-administration and may prevail for months after the completion of the treatment (Banach et al., 2017). Interestingly, flavonoids are known to propagate the anticancer activity of cisplatin through different mechanisms, including anti-inflammatory and antioxidant propensities, while mitigating its adverse effects (Maatouk et al., 2020; Din et al., 2019).

Anxiety, on the other hand, is imposed on patients by both the nature of the disease and as an adverse effect of the treatment. This may compromise the patient’s wellbeing, both physically and mentally (Hung et al., 2021). Human brains consider certain elements as a threat to homeostasis and react by invoking stress against these stressors. To maintain homeostasis, all the body machinery, including the sensory, endocrine, autonomic, and immune systems, comes into action. This condition, if not treated in time, may manifest as anxiety and depression. One study reported the advent of stress-related symptoms in cancer survivors, which ultimately deteriorated their quality of life (Miaskowski et al., 2018). Most patients do not consider stress to be a matter of concern, while others seek medical advice and are placed on multi-drug therapy for treating anxiety and depression along with chemotherapy. It has been reported that patients with chronic neuropathic pain are prescribed opioids, anti-depressants, or anticonvulsants (Sanjida et al., 2016), adding several co-morbidities to the existing treatment regimen.

6-HF significantly attenuated anxiety-like behavior in the male rats, as ascertained by the increased open-arm entries, time spent in the open arm and at the center of EPM, and the enhanced wall-rearing and head-dipping frequencies comparable to diazepam. Moreover, the anxiolytic-like actions were further confirmed in two other tests, the staircase and the open-field test. The standard drug diazepam showed anxiolytic-like propensity in all tests; however, it showed no effect on the time spent at the center of the open field, which is in alignment with studies previously reported. It is reported that drugs affecting the core body temperature, like diazepam, can reduce the time spent at the center of the open-field apparatus (Jimenez et al., 2023). The incidence of anxiety allied with neuropathic pain in rat models has also been reported, suggesting that chronic neuropathic pain results in the emergence of anxiety, which further exaggerates pain perception (Röska, 2009). The existence of anxio-depressive consequences of neuropathic pain in different animal models of neuropathy is well documented (Roeska et al., 2008; Li et al., 2014). In this study, the anti-allodynic and anxiolytic-like actions of 6-HF were evaluated in a rat model. The evaluation of the two parameters allied with chemotherapy in a similar animal model remains a limitation of this research, and it could be a prospective extension of the present study.

The decrease in the nociception behavior toward mechanical stimuli by 6-HF may be related to its positive allosteric modulation at the GABA-A and opioid receptors, as previously reported in our laboratory (Akbar et al., 2023). It is evident from the research on flavonoids that they are allosteric modulators of the GABA-A receptors (Syafni et al., 2022; Hall et al., 2014). In this regard, 6-methoxyflavanone, having a methoxy substitution at position six of the flavanone ring-A, has been reported to exert positive allosteric modulation at the human recombinant α1β2γ2L and α2β2γ2L GABA_A_ receptors expressed in *Xenopus laevis* oocytes (Hall et al., 2014). However, the anxiolytic-like activity of 6-HF may be attributed to its potential interaction with the α_2_-subunit or α5-subunit containing GABA-A receptors. These receptors are considered to be involved in the pathophysiology of anxiety (Chen et al., 2019).

The neuroprotective effect of 6-HF can also be associated with its anti-inflammatory effect (Akbar et al., 2023). The accumulation of cisplatin in the dorsal root ganglia stimulates the production of pro-inflammatory cytokines (IFN-γ and IL-1β), resulting in increased neuropathic pain symptoms (Kleckner et al., 2021). The activation of microglial cells and macrophages as a result of the release of pro-inflammatory cytokines further exaggerates chronic pain or sensory deficits (Domingo et al., 2022).

Citrus flavanones have been the center of focus for neurologists for the past few decades. The growing interest in this field is attributed to the potential of these molecules to act upon target sites involved in pain modulation, such as opioids and GABA-A receptors, and decrease the pro-inflammatory mediators (Rao et al., 2021). Thus, 6-HF, previously reported as having a significant anti-allodynic and vulvodynic effect in a diabetes-induced neuropathy model (Akbar et al., 2023), was found to have significance in treating pain allied with neurotoxicity induced by cisplatin. This effect of 6-HF can be attributed to the decrease in the nociceptor sensitization via acting upon GABA-A/opioid receptors and a decrease in the release of pro-inflammatory mediators. Moreover, the anxiolytic-like actions of 6-HF may be related to its activity over the α_2_-subunit or α5-subunit containing GABA-A receptors. However, further studies are required to confirm the mechanism through which 6-HF provides neuroprotection against the platinum analogs and the suppression of anxiety-like behavior.

## 6 Conclusion

Co-administration of 6-HF with the platinum analogs used as chemotherapeutic agents in treating different types of cancers may provide neuroprotection against the neurotoxic effects of these agents. Additionally, 6-HF may also reduce the anxiety-like effects of chemotherapy although further research is needed to observe both parameters in the same animal model.

## Data Availability

The original contributions presented in the study are included in the article/supplementary material; further inquiries can be directed to the corresponding author.

## References

[B1] Ahmad N. Subhan F. Islam l. Shahid M. Ullah N. Ullah R. (2021). A novel gabapentin analogue assuages neuropathic pain response in chronic sciatic nerve constriction model in rats. Behav. Brain Res. 405, 113190. 10.1016/j.bbr.2021.113190 33607164

[B2] Akbar S. Subhan F. Akbar A. Habib F. Shahbaz N. Ahmad A. (2023). Targeting anti-inflammatory pathways to treat diabetes-induced neuropathy by 6-hydroxyflavanone. Nutrients 15 (11), 2552. 10.3390/nu15112552 37299516 PMC10255489

[B3] Akbar S. Subhan F. Karim N. Aman U. Ullah S. Shahid M. (2017). Characterization of 6-methoxyflavanone as a novel anxiolytic agent: a behavioral and pharmacokinetic approach. Eur. J. Pharmacol. 801, 19–27. 10.1016/j.ejphar.2017.02.047 28257822

[B4] Authier N. Gillet J.-P. Fialip J. Eschalier A. Coudore F. (2003). An animal model of nociceptive peripheral neuropathy following repeated cisplatin injections. Exp. Neurol. 182 (1), 12–20. 10.1016/s0014-4886(03)00003-7 12821373

[B5] Banach M. Juranek J. K. Zygulska A. L. (2017). Chemotherapy‐induced neuropathies—a growing problem for patients and health care providers. Brain Behav. 7 (1), e00558. 10.1002/brb3.558 28127506 PMC5256170

[B6] Blackadar C. B. (2016). Historical review of the causes of cancer. World J. Clin. Oncol. 7 (1), 54–86. 10.5306/wjco.v7.i1.54 26862491 PMC4734938

[B7] Bray F. Ferlay J. Soerjomataram I. Siegel R. L. Torre L. A. Jemal A. (2018). Global cancer statistics 2018: GLOBOCAN estimates of incidence and mortality worldwide for 36 cancers in 185 countries. CA a cancer J. Clin. 68 (6), 394–424. 10.3322/caac.21492 30207593

[B8] Chaplan S. R. Bach F. Pogrel J. Chung J. Yaksh T. (1994). Quantitative assessment of tactile allodynia in the rat paw. J. Neurosci. methods 53 (1), 55–63. 10.1016/0165-0270(94)90144-9 7990513

[B9] Chen X. van Gerven J. Cohen A. Jacobs G. (2019). Human pharmacology of positive GABA-A subtype-selective receptor modulators for the treatment of anxiety. Acta Pharmacol. Sin. 40 (5), 571–582. 10.1038/s41401-018-0185-5 30518829 PMC6786312

[B10] Choi D. W. Kang H. Zhang H. S. Jhang H. Jeong W. Park S. (2023). Association of polypharmacy with all‐cause mortality and adverse events among elderly colorectal cancer survivors. Cancer 129 (17), 2705–2716. 10.1002/cncr.34813 37118834

[B11] Din Z. U. Iftikhar S. Rehman Z. Harris M. Ambreen S. Khattak M. (2019). Effect of 6-hydroxyflavone oncisplatininduced histopathological and biochemical changes in liver of sprague-dawely rats. J. Khyber Coll. Dent. 9 (04), 96–102. 10.33279/jkcd.v9i04.405

[B12] Djordjević M. Ilić J. Stojanovic N. M. (2023). *CISPLATIN-AN overview of its efficiency and toxicity.* Facta universitatis. Ser. Med. Biol., 025–035. 10.22190/FUMB230122002D

[B13] Domingo I. K. Latif A. Bhavsar A. P. (2022). Pro-inflammatory signalling PRRopels cisplatin-induced toxicity. Int. J. Mol. Sci. 23 (13), 7227. 10.3390/ijms23137227 35806229 PMC9266867

[B15] Hall B. J. Karim N. Chebib M. Johnston G. A. Hanrahan J. R. (2014). Modulation of ionotropic GABA receptors by 6-methoxyflavanone and 6-methoxyflavone. Neurochem. Res. 39, 1068–1078. 10.1007/s11064-013-1157-2 24078264

[B16] Hall C. S. (1934). Emotional behavior in the rat. I. Defecation and urination as measures of individual differences in emotionality. J. Comp. Psychol. 18 (3), 385–403. 10.1037/h0071444

[B17] Han F. Y. Wyse B. D. Smith M. T. (2014). Optimization and pharmacological characterization of a refined cisplatin-induced rat model of peripheral neuropathic pain. Behav. Pharmacol. 25 (8), 732–740. 10.1097/FBP.0000000000000090 25325291

[B18] Hung H.-W. Liu C.-Y. Chen H.-F. Chang C.-C. Chen S.-C. (2021). Impact of chemotherapy-induced peripheral neuropathy on quality of life in patients with advanced lung cancer receiving platinum-based chemotherapy. Int. J. Environ. Res. Public Health 18 (11), 5677. 10.3390/ijerph18115677 34073174 PMC8199281

[B19] Ibrahim E. Y. Ehrlich B. E. (2019). Prevention of chemotherapy-induced peripheral neuropathy: a review of recent findings. Crit. Rev. oncology/hematology 145, 102831. 10.1016/j.critrevonc.2019.102831 PMC6982645 31783290

[B20] Jimenez J. A. McCoy E. L. David F. Z. Mark J. (2023). The open field assay is influenced by room temperature and by drugs that affect core body temperature. F1000Research 12, 234. 10.12688/f1000research.130474.3 38863500 PMC11165296

[B21] Kleckner I. R. Jusko T. A. Culakova E. C. Kaitlin K. Amber S. A. Matthew I. (2021). Longitudinal study of inflammatory, behavioral, clinical, and psychosocial risk factors for chemotherapy-induced peripheral neuropathy. Breast cancer Res. Treat. 189, 521–532. 10.1007/s10549-021-06304-6 34191201 PMC8668235

[B22] Li Q. Yue N. Liu S.-B. Wang Z.-F. Mi W.-L. Jiang J.-W. (2014). Effects of chronic electroacupuncture on depression-and anxiety-like behaviors in rats with chronic neuropathic pain. Evidence-Based Complementary Altern. Med. 2014, 158987. 10.1155/2014/158987 PMC3984799 24795763

[B23] Maatouk M. Abed B. B. Krifa M. Khlifi R. Ioannou I. Ghedira K. (2020). Heat treatment and protective potentials of luteolin-7-O-glucoside against cisplatin genotoxic and cytotoxic effects. Environ. Sci. Pollut. Res. 27, 13417–13427. 10.1007/s11356-020-07900-7 32026362

[B24] Macri S. Adriani W. Chiarotti F. Laviola G. (2002). Risk taking during exploration of a plus-maze is greater in adolescent than in juvenile or adult mice. Anim. Behav. 64 (4), 541–546. 10.1006/anbe.2002.4004

[B25] McNeish B. L. Richardson J. K. Whitney D. G., Chemotherapy induced peripheral neuropathy onset increases the early risk for depression and anxiety in breast cancer survivors *.* (2021). 10.1111/ecc.13648 35830192

[B26] Miaskowski C. Paul S. M. Mastick J. Abrams G. Topp K. Smoot B. (2018). Associations between perceived stress and chemotherapy-induced peripheral neuropathy and otoxicity in adult cancer survivors. J. pain symptom Manag. 56 (1), 88–97. 10.1016/j.jpainsymman.2018.02.021 PMC6015523 29524582

[B27] Nightingale G. Skonecki E. Boparai M. K. (2017). The impact of polypharmacy on patient outcomes in older adults with cancer. Cancer J. 23 (4), 211–218. 10.1097/PPO.0000000000000277 28731943

[B28] Pellow S. Chopin P. File S. E. Briley M. (1985). Validation of open: closed arm entries in an elevated plus-maze as a measure of anxiety in the rat. J. Neurosci. methods 14 (3), 149–167. 10.1016/0165-0270(85)90031-7 2864480

[B29] Rao P. N. Mainkar O. Bansal N. Rakesh N. Haffey P. Urits I. (2021). Flavonoids in the treatment of neuropathic pain. Curr. Pain Headache Rep. 25 (7), 43. 10.1007/s11916-021-00959-y 33961144

[B30] Rodgers R. Cole J. Aboualfa K. Stephenson L. (1995). Ethopharmacological analysis of the effects of putative ‘anxiogenic’agents in the mouse elevated plus-maze. Pharmacol. Biochem. Behav. 52 (4), 805–813. 10.1016/0091-3057(95)00190-8 8587923

[B31] Roeska K. Doods H. Arndt K. Treede R.-D. Ceci A. (2008). Anxiety-like behaviour in rats with mononeuropathy is reduced by the analgesic drugs morphine and gabapentin. Pain 139 (2), 349–357. 10.1016/j.pain.2008.05.003 18565660

[B32] Röska K. (2009). Relationship between pain and anxiety in rats. Dissertation. Mainz. 10.25358/openscience-1591

[B33] Sanjida S. Janda M. Kissane D. Shaw J. Pearson S. A. DiSipio T. (2016). A systematic review and meta‐analysis of prescribing practices of antidepressants in cancer patients. Psycho‐Oncology 25 (9), 1002–1016. 10.1002/pon.4048 26775715

[B34] Shahid M. Subhan F. Islam N. U. Ahmad N. Farooq U. Abbas S. (2020). The antioxidant N-(2-mercaptopropionyl)-glycine (tiopronin) attenuates expression of neuropathic allodynia and hyperalgesia. Naunyn Schmiedeb. Arch. Pharmacol. 394, 603–617. 10.1007/s00210-020-01995-y 33079239

[B35] Shukla R. Vikas V. Gautam P. L. Santram P. (2019). “Role of flavonoids in management of inflammatory disorders,” in Bioactive food as dietary interventions for arthritis and related inflammatory diseases (Elsevier), 293–322.

[B36] Simiand J. Keane P. Morre M. (1984). The staircase test in mice: a simple and efficient procedure for primary screening of anxiolytic agents. Psychopharmacology 84, 48–53. 10.1007/BF00432023 6149594

[B37] Syafni N. Faleschini M. T. Garifulina A. Danton O. Gupta M. P. Hering S. (2022). Clerodane diterpenes from casearia corymbosa as allosteric GABAA receptor modulators. J. Nat. Prod. 85 (5), 1201–1210. 10.1021/acs.jnatprod.1c00840 35475609 PMC9150179

[B38] Tang M. Li X. (2020). Adverse reactions of antidepressant drugs and their application in patients with cardiovascular diseases. Zhong nan da xue xue bao. Yi xue ban= J. Central South Univ. Med. Sci. 45 (10), 1228–1233. 10.11817/j.issn.1672-7347.2020.190160 33268585

[B39] Thouaye M. Yalcin I. (2023). Neuropathic pain: from actual pharmacological treatments to new therapeutic horizons. Pharmacol. and Ther. 251, 108546. 10.1016/j.pharmthera.2023.108546 37832728

[B40] Walia V. Garg C. Garg M. (2019). Lithium potentiated, pyridoxine abolished and fluoxetine attenuated the anxiolytic effect of diazepam in mice. Brain Res. Bull. 150, 343–353. 10.1016/j.brainresbull.2019.06.008 31201833

[B41] Zhou Y. Cai S. M. Aubin Y. Jie G. Kimberly L. Shan Z. P. (2019). The natural flavonoid naringenin elicits analgesia through inhibition of NaV1. 8 voltage-gated sodium channels. ACS Chem. Neurosci. 10 (12), 4834–4846. 10.1021/acschemneuro.9b00547 31697467

